# A Case Driven Study of the Use of Time Series Classification for Flexibility in Industry 4.0

**DOI:** 10.3390/s20247273

**Published:** 2020-12-18

**Authors:** Julien Polge, Jérémy Robert, Yves Le Traon

**Affiliations:** Interdisciplinary Centre for Security, Reliability and Trust, University of Luxembourg, 6 Rue Richard Coudenhove-Kalergi, L-1359 Luxembourg, Luxembourg; jeremy.robert@uni.lu (J.R.); yves.letraon@uni.lu (Y.L.T.)

**Keywords:** automation architecture, flexibility, Industry 4.0, machine learning, time series classification

## Abstract

With the Industry 4.0 paradigm comes the convergence of the Internet Technologies and Operational Technologies, and concepts, such as Industrial Internet of Things (IIoT), cloud manufacturing, Cyber-Physical Systems (CPS), and so on. These concepts bring industries into the big data era and allow for them to have access to potentially useful information in order to optimise the Overall Equipment Effectiveness (OEE); however, most European industries still rely on the Computer-Integrated Manufacturing (CIM) model, where the production systems run as independent systems (i.e., without any communication with the upper levels). Those production systems are controlled by a Programmable Logic Controller, in which a static and rigid program is implemented. This program is static and rigid in a sense that the programmed routines cannot evolve over the time unless a human modifies it. However, to go further in terms of flexibility, we are convinced that it requires moving away from the aforementioned old-fashioned and rigid automation to a ML-based automation, i.e., where the control itself is based on the decisions that were taken by ML algorithms. In order to verify this, we applied a time series classification method on a scale model of a factory using real industrial controllers, and widened the variety of parts the production line has to treat. This study shows that satisfactory results can be obtained only at the expense of the human expertise (i.e., in the industrial process and in the ML process).

## 1. Introduction

Over the last few years, the manufacturing sector has been facing a new industrial revolution. This fourth industrial revolution—Industry 4.0—originates from Germany, and was initially a proposal for developing a new concept of German economic policy in 2011 [1]. Enabled by different technologies and concepts, such as the Internet-of-Things (IoT), data-mining, and Machine Learning (ML) algorithms, new decision-making processes, Cyber-Physical Systems (CPS), Operational and Information Technologies convergence (OT/IT) [2], the main features of the Industry 4.0 are the following [3,4,5,6,7]:

- Customisation/Individualisation of the products: The production needs to adapt to the customer’s requirements that tend to be increasingly precise and individual. This also allows for the development of innovative business models.
- Flexibility: The production chain needs to automatically adapt itself to the evolving requirements (e.g., the range of products it should produce or the speed of the conveyer), to enable productivity gains.
- Product traceability: one needs to be able to identify all of the processes through which raw material, parts, and products have been modified/realised/assembled.
- Optimisation of the production process: thanks to the IoT devices and the information gathered and analysed by machine learning algorithms, it is possible to have a precise overview of the production process and, thus, optimise resources and, for example, predict forthcoming maintenance actions.

Meeting these expectations requires that European manufacturing companies rely on more advanced ICT bases and integrate IoT (or IIoT) at a larger scale, as it is considered to be a key enabler of Industry 4.0 [1]. IoT—which describes the network of physical objects embedding sensing capabilities and software components in order to exchange data over the internet—is, in fact, an overarching term that applies to all of the sectors that can be digitised using physical sensors. IoT thus encompasses many of the current and future technologies and concepts [8], such as smart cities where different devices (e.g., sensors, cameras, etc.) are installed in a city in order to monitor the vehicular traffic, measure and report the environmental pollution, provide information on the weather, guide the car drivers into parking lots with the most space available, and so on [9]. Another important area of IoT applications is smart grids. Smart grids are electrical power grids that are smarter than traditional power grids thanks to IoT technologies. These smart grids self-heal, distribute electricity on demand, are monitored and controlled in real-time, and they rely on several small power producers, which makes the grids more efficient than traditional grids [10,11,12]. IoT is also used in healthcare, for example, to monitor patients’ health conditions [13], by collecting different information, such as electrocardiogram (ECG), blood pressure, etc. It allows for reducing the risk of errors and, thus, improving the efficiency and lowering the costs of healthcare services [14]. Industry 4.0/Smart manufacturing falls in the list of sectors that are covered by IoT, and, since it heavily relies on IoT technologies as enablers. IoT technologies allow for new applications and improvements in the manufacturing world, such as IoT-based cloud manufacturing, cyber-physical manufacturing, energy efficiency management, supply chain, and logistics traceability improvements, as reported in [15].

Unfortunately, most European industries—especially companies having decades of existence—do not have up-to-date ICT and automation infrastructures. Indeed, current manufacturing systems still rely on the Computer-Integrated Manufacturing (CIM) model, where the production systems run as independent systems (i.e., without any communication with the upper levels). The brain of such production systems is the programmable logic controller (PLC), which is an industrial computer monitoring its inputs (sensors) and outputs (actuators) in a cyclic manner. It makes decisions by itself based on the internal program logic. Therefore, this program is static and rigid in a sense that the programmed routines cannot evolve over the time unless a human modifies the PLC program. It makes difficult or almost impossible, for instance, to widen the range of products manufactured on a same line without having to stop it and load another program each time that we change the range of products. It would be the same difficulty for all major functional or environmental changes, e.g., a variation of the production speed or a move of the production system to a place where the lighting environment is different would require a long manual configuration phase. So far, this is achieved by blue-collar workers (i.e., the operators) that monitor, operate, and maintain, in operational condition, the production lines.

Working together with an automotive parts manufacturer—Cebi Luxembourg S.A.—through an R&D partnership, our main objective is to improve the overall efficiency of the production systems while shortening the “Measure Decision Act” loop. Thanks to a lot of sensor data (also known as time-series (TS)) being collected, ML algorithms can ingest them for monitoring the factory’s overall performance in real time, as well as understand and predict technical issues. However, in order to further improve the flexibility, we are convinced that it requires moving away from the aforementioned old-fashioned and rigid automation to a ML-based automation, i.e., where the control itself is based on the decisions that were taken by ML algorithms. We propose exploring whether and to what extent such a ML-based automation approach can replace a manual and static setting of a production line configuration (i.e., where all of the parameters have been manually set for the limited range of products).

Therefore, the contributions are threefold: (i) we concisely review time-series classification methods and their applications in the industry, (ii) we apply a selected time-series classification method from the literature to a real use-case developed on a scale model of a factory (FischerTechnik factory simulation) relying on a real ICT infrastructure (detailed in the next section), as it is not possible to stop a production line for deploying such a scenario without beforehand demonstrating the feasibility, and (iii) we discussed the implications and limitations of using such an approach from a technical point of view in the industry, to serve as knowledge based on how to evolve towards such an approach in real-settings.

The rest of this paper is organised, as follows: Section 2 explains the problem, Section 3 gives a review on time-series classification methods and their implementation in the industry, Section 4 presents our case study and experimental setup. Experiments have been conducted in order to improve the flexibility of production lines and the results are presented in Section 5. Section 6 discusses our results and methods. Additionally, finally, Section 7 concludes the paper and presents ideas for the future.

## 2. Problem Statement

**Figure 1:**
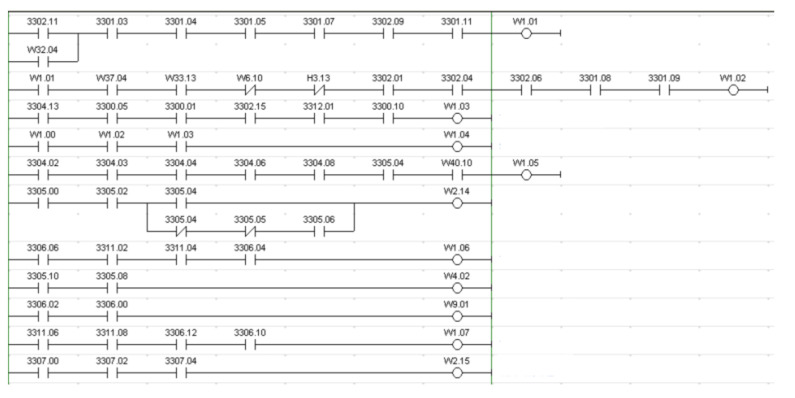
Ladder program from a traditional controller.

Our main goal is to improve the flexibility of the production lines, i.e., the ability of the line to automatically adapt itself to evolving requirements (a larger variety of parts to produce, for example). However, European manufacturing companies usually rely on the CIM model. Consequently, each production system has its own logic that is implemented in a PLC. PLC programs are developed in a rigid manner, and they are not meant to evolve in order to adapt to a changing environment. Any modification to the code requires human intervention. This old-fashioned and rigid automation model is a hurdle to the improvement of the production lines’ flexibility. As an example,  Figure 1 is an excerpt from a PLC program of a real production line (the whole program contains over 6000 instructions, each line is an instruction). Modifying this program requires having a perfect understanding of the process and the program, since the sequence of instructions is important and it should not be randomly modified (it could impact the process). Each modification implies a large testing process on the production line.

Consequently, to tackle the limitations of static programs, we propose exploring to what extent these static PLC programs could be replaced by a state-of-the-art ML-based method. To do so, we first need a safe development environment, in which we are able to experiment and test without damaging anything or modifying the processes in the factory. Indeed, the industrial partner cannot afford the downtime that it would require to test this solution; he needs to understand the benefits of using such a method and evaluating the Return On Investment (ROI), and finally, we can also propose a more innovative architecture using new generation PLCs in a development environment. Another key factor in the choice of our development environment is its use in scientific papers. Finally, we need to find a use case that complies with several properties: (i) it should be representative, i.e., common in the industry, such as an anomaly detection process, a sorting process, or more generally a classification process, (ii) it should be minimalist and canonical, i.e., based on one variable, if it works with one variable and a small amount of data (which is a limit case for ML), it will most likely work with more variables.

The *FischerTechnik* 24 V factory simulation (FischerTechnik Factory simulation website, https://www.fischertechnik.de/en/service/elearning/simulating/fabrik-simulation-24v) has been chosen. This platform has been used in the literature for different objectives, such as CPS security research hackathons [16], in order to develop a new low-cost, but powerful, controller [17], or to develop a multi-agent control system [18], and the automation architecture (controllers, sensors, and actuators) is representative of a real production line, since it relies on real industrial controllers to control the different stations that compose the platform (our architecture is detailed in Section 4). The four stations are: (i) the automated warehouse where parts are stored, (ii) the multi-processing station (simulating an oven and a saw), (iii) the vacuum gripper robot that is used to move the parts between the warehouse and the multi-processing station, and (iv) the sorting line with the colour sensor that is used to sort the parts according to their colour.

**Figure 2:**
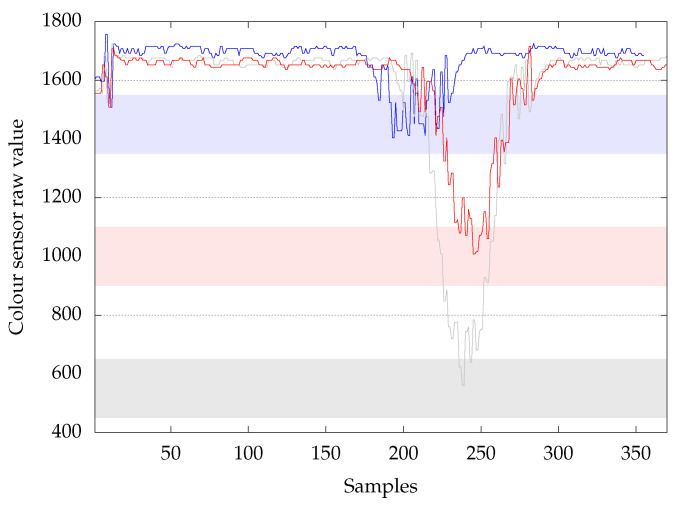
Time series of the three colours & thresholds.

On this platform, we focused on the fourth station, namely the sorting line. This choice is justified by the fact this station is the one that best fits our case by using a colour sensor returning numeric values, on which a decision is taken under temporal constraints. Thus, this case is more interesting, while the other stations rely on position sensors or limit switches, which return binary values. As a matter of fact, it is composed of three sensors: An input sensor, a colour sensor, and an output sensor, and three actuators to store the part in the right stock according to its colour. Based on those inputs/outputs, the program logic implemented in the controller is as follows: The value of the colour sensor is collected every 10 ms from the moment the part is detected by the input sensor, until the moment the part is detected by the output sensor. By doing so, we are able to create time series, such as the ones depicted in  Figure 2, where the sizes range from 350 to 500 observations per TS due to the fact that parts are going from a conveyer belt to another, and this could take more or less time. The blue TS is from a blue part, the red TS is from a red part, and the grey TS is from a white part (the platform comes with blue, white, and red parts).

This is a traditional time series classification (TSC) case. The next section gives a background on TSC methods and their application in industry.

## 3. Background & Related Work

### 3.1. Time Series Classification

An univariate time series (TS) is an ordered sequence of values that were taken by the same variable, recorded at fixed sampling rate. Time series are increasingly generated and analysed in different application domains, such as finance (e.g., stock markets’ fluctuations), health monitoring applications (e.g., electrocardiograms), meteorology (e.g., weather forecasting), and industry (e.g., sensor values) [19,20,21,22]. The multiplication of application areas is driven by, among other things, the increasing use of sensors and IoT devices in several domains, as denoted in the previous subsection (e.g., 61.8% of the UCR datasets are sensor data or simulated data) and the fact that increasing researchers and practitioners are using time series to model non-temporal observations [21] (e.g., 38.2% of the UCR datasets are data from images).

The objective of time series classification (TSC) is to assign a class label to a TS that is based on a set of predefined classes. Basically, a set of labelled TS is learned by an algorithm, and the algorithm applies what it learned to label each non-labelled TS of a testing set. The TS classifiers can be divided into three categories:

- Whole series similarity-based methods [23]: These methods measure similarity or dissimilarity (distance) between two entire TS and usually use a 1-NN classifier in order to evaluate the distance. The most common methods are 1-NN Euclidian distance (ED) and 1-NN Dynamic Time Warping (DTW [24]) [25]. Several other algorithms have also been proposed, such as Weighted DTW (WDTW), Time warp edit (TWE), or Complexity invariant distance (CID), as denoted in the review that was written by A. Bagnall et al. [23].
- Feature-based/Symbolic representation-based methods: These methods usually compute features from the TS and then compare the features that were computed from the training TS to the features computed from the test TS. These methods can be subdivided into two types: shapelets-based methods and Bag-Of-Patterns (BOP)-based methods. Shapelets, as introduced by L. Ye & E. Keogh [26], are subsequences of TS that are the most representative of a class. Subsequently, J. Lines et al. proposed a Shapelet Transform (ST) [27] in order to improve the classification accuracy by extracting the the best *k* shapelets for each class and avoid overfitting. The result of this transform can be used as an input of traditional classifiers. Several other shapelets-based methods exist, such as Fast Shapelets (FS) or Learning Shapelets (LS).
  On the other hand, Bag-Of-Patterns (BOP) is a histogram-based similarity measure, which is similar to Bag-Of-Words for text data [28]. BOP uses a sliding window to extract subsequences of the TS of a fixed length, and then each subsequence is converted into a Symbolic Aggregate approXimation [29] (SAX) string. The most accurate classifier for combining with BOP-SAX is 1-NN using Euclidian distance [30]. P. Senin & S. Malinchik proposed the Symbolic Aggregate approXimation-Vector Space Model (SAX-VSM), which uses a different feature weighting system from BOP-SAX [30]. P. Schafer introduced the Bag-of-SFA-Symbols (BOSS) model [31], which uses the Symbolic Fourier Approximation (SFA) [32] instead of SAX. P. Schafer & U. Leser then presented the Word ExtrAction for the time Series cLassification (WEASEL) [22] method, which is a BOP-based method that uses a specific method in order to derive features, so as to be fast enough to satisfy the requirements of sensor-driven application and still give accurate classification results. WEASEL will be further explained in the next section. Note that Bag-Of-Patterns-based methods have a linear complexity.
- Ensemble classifiers: These methods use a set of classifiers and aggregate the results while using different methods to return a classification result. Ensemble methods are the most accurate methods, yet they are computationally very expensive, since each classifier inside the ensemble has to be run. J. Lines & A. Bagnall proposed the Elastic Ensemble [33] (EE), which is an ensemble including elastic measures, such as DTW, variants of DTW, and ED. The same authors presented the Collective of Transformation-Based Ensemble (COTE) [34], which is an ensemble of 35 classifiers. The authors then proposed HIVE-COTE in 2016 [35], which is an improvement of COTE while using a hierarchical probabilistic voting structure.

The next subsection presents the review on the application of such TSC in the industry.

### 3.2. Time Series Classification in Industry

With the *Industry 4.0* paradigm comes the convergence of the Internet Technologies and Operational Technologies, and concepts, such as Industrial Internet of Things (IIoT) (which is the IoT applied in an industrial environment), cloud manufacturing, and so on. These concepts and the ever-growing number of deployed sensors [22] are pushing industries into the big data age [36]. To exploit the full potential of this increasing quantity of data, practitioners are more and more interested in deploying ML-based methods which will help them to analyse the data and extract useful information related to the processes, the equipment, the production and so forth. However, this is not limited to manufacturing and it can be extended to other fields, such as smart-grid [37,38], smart-cities [9], transportation [39], health, sport, etc.

There are plenty of papers in the literature whose authors are working on implementing time series classification in industry on different use cases.  Table 1 references those papers and summarises the approach used, whether it is a traditional ML-based or deep learning (DL)-based approach, the kind of data they use, and the area of application. We focused on papers on manufacturing and application domains that could have to deal with the same problems, and detailed what they do in the field (e.g., anomaly detection, health management, and so on).

To wrap-up this part of the related work, the trend seems to be the following: The papers using univariate TS are more focused on classification, monitoring, and anomaly detection, generally descriptive analysis and use ML-based methods. More complex problems, including prediction, often use ML-based approaches and multivariate TS or fusion data. Finally, prognostics and health management problems tend to use deep learning-based methods and multivariate TS. Deep learning methods are becoming more common for time series classification, but these methods are used as a black box. In our case, provided that we want to preserve the expert knowledge and, when considering our application, we will focus our interest on ML-based methods for univariate time series classification and, more precisely, the WEASEL method.

### 3.3. WEASEL

In order to use time series classification methods in our case study, we needed a method that is able to deal with TS with variable lengths, which are sometimes noisy, because they are issued by an inaccurate sensor (in our case, but it could also be due to the dust or luminosity in a real industrial environment); additionally, the classification time should be fast enough to still be able to sort out the parts in the right stock.

WEASEL has been proposed for addressing the challenges that are associated with sensor-driven applications [22]; namely, this method is robust to noise, able to scale to the number and the length of TS, able to classify TS with different sizes and different offsets, and does not need to know *a priori* the structure of the characteristic pattern. According to this description, this method *a priori* fulfills our requirements for such a case study. That is the primary reason why we select and apply this method in this paper.

In addition, WEASEL is a great trade-off between good accuracy and fast training and prediction time. WEASEL was the second most accurate method after the COTE ensemble, but, when it comes to training time and prediction time, the few classifiers that are faster than WEASEL are less accurate, as demonstrated by the authors by comparing their method against the best core classifiers on all the UCR datasets [22]. This feature (prediction time) is important in our case, and it constitutes a second reason to select the WEASEL method.

WEASEL uses normalised sliding windows of different sizes in order to decompose the TS, and then it approximates each window while using the Fourier transform. Subsequently, the statistical ANOVA F-test is applied to keep the Fourier coefficients that allow for differentiating the TS from different classes. Those kept coefficients are then discretised into words while using information gain binning. A Bag-Of-Pattern is built from these words and encodes unigrams, bigrams, and the windows. The Chi-Squared test is applied to the Bag-Of-Pattern to reduce the feature space and only keep the most discriminative words. Finally, the classification is done by logistic regression.

To the best of our knowledge, only a few works have been done while using WEASEL. M. Middlehurst et al. [63] evaluated WEASEL to find out whether it could be a good replacement for BOSS in the HIVE-COTE ensemble. The authors compared the WEASEL, BOSS, and BOSS variants; it turns out that WEASEL is the most accurate method, yet it is the slowest to build on average. W. Sousa Lima et al. applied BOSS, BOSS-Vector Space (BOSS-VS), SAX-VSM, and WEASEL on human activity data sets from three databases and compared them regarding the accuracy, the processing time, and the memory consumption [64]. Even if WEASEL has never been implemented in an industrial context, it seems to be promising for our use-case, and it is selected for discussing an innovative and flexible automation based on traditional ML. Note that the method is not compared with other ML/TSC methods in this paper, but only with the most effective and efficient one at the time of the study. For a comparative study, we redirect to the one that has been conducted in [22], which demonstrates that WEASEL outperforms a baseline of ML algorithm for TS classification. In this paper, building on the best existing algorithm that is found in the literature, the goal is to study to what extent it can be applied successfully, the limits in using it, and the adaptations (here preprocessing) that are required to optimise it to a typical Industry 4.0 problem. We believe that this would be a natural and intuitive choice to take the “best” algorithm in any real-world setting.

The originality of this work is in the fact that we design a more flexible automation architecture, integrating a TSC method from the literature, which we evaluate by conducting live tests. By conducting live tests, we are able to demonstrate the applicability of such a method in an industrial production process, in order to show that this method allows a more flexible production and to highlight the limits and adaptations that are required to implement such a method.

## 4. On the Use of ML in the Automation Architecture

European manufacturing companies usually rely on the CIM model, where each production system has it own logic implemented in a PLC, as mentioned in Section 2. Such programs are often developed in a cyclic manner; i.e., by successively reading sensors’ inputs, processing them, and finally updating outputs (actuators). There is no (or very few) intelligence, which makes them rigid. To make them a bit more flexible, one way would be to develop functions that would be triggered by API calls to create the current logic. This would also enable introducing external ML-based programs while keeping industrial PLCs in the automation architecture. To go even further, those PLCs could be replaced by industrial (micro) computers having more resources for using more advanced/intelligent functionalities. In that sense, we designed the architecture of our *FischerTechnik* factory simulation, so as to demonstrate this possible evolution.

It consists of three controllers/PLCs that possess their own sets of inputs/sensors and outputs/actuators. Each PLC implements its own program logic by using the programming languages that are related to the hardware. For instance, a Function Block Diagram and/or Sequential Flow Chart is used on the Crouzet em4 (Crouzet website, https://automation.crouzet.com/em4-nano-plc/) for controlling the warehouse. Note that this PLC implements several functions that are triggered by a central controller while using the Modbus/TCP protocol. For controlling the robot, we used a Controllino Maxi PLC (Controllino website, https://www.controllino.biz/product/controllino-maxi/) (based on arduino) and, for the multi-processing station and the sorting line, we used two Wago EtherCAT slaves with an EtherCAT master that was implemented on a Raspberry PI3. Those latter use C-based programs, and they have been chosen to show the evolution in terms of automation architecture. First, we defined a manual and static setting of a production line configuration for the sorting line. This represents the type of static programs implemented in old-fashioned controllers. The next section will provide more details.

Let us remind that our objective is to advance from a static setting of the program (such as shown in Algorithm 1, where the variable *ts* is a vector of n integer values v_j_ and *th*_i_ are integer thresholds (i = 1, 2, 3, 4, 5, 6)). Accordingly, instead, the idea is to have a dedicated agent that is capable of predicting/classifying a time-series according to a trained model (that can evolve easily over the time). In that architecture, the controller can implement the program logic that is defined in Algorithm 2, where *ts* is a vector of n integer values v_j_ and where line 3 is an API call to this dedicated ML-based agent. Note that this agent can run directly on the same device or another (in a distributed architecture).
**Algorithm 1** Static program.1:ts← [v1,v2,…,v_n_]2:minTs← min(ts)3:**if** th_1_ < minTs < th_2_ **then**4: colour← blue5:**else if** th_3_ < minTs < th_4_ **then**6: colour← white7:**else if** th_5_ < minTs < th_6_ **then**8: colour← red9:**end if**10:sort(colour)
**Algorithm 2** Flexible program using ML prediction.1:ts← [v1,v2,…,v_n_]2:colour← getPrediction(ts)3:sort(colour)

**Figure 3:**
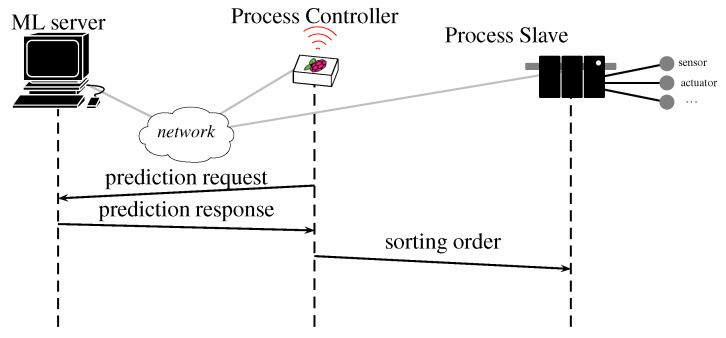
Scenario architecture.

In our testbed, this agent uses WEASEL and its “official” Java implementation v.3. It was implemented on a HTTP server that was hosted by a MacBook Pro with a 2.8 GHz Intel Core i7 processor. When the values are collected and the TS is generated, it is sent to the server by an HTTP client (as part of the main program logic) on the Raspberry PI3 (depicted as the prediction request in Figure 3), then the server executes WEASEL on the TS and sends its class prediction (depicted as prediction response in Figure 3) and, based on the class, the controller sends the command to the actuators to push the part in the right stock (depicted as sorting order in Figure 3). Thereby, the colour verification and the sorting are done “on-the-fly”.

**Figure 4:**
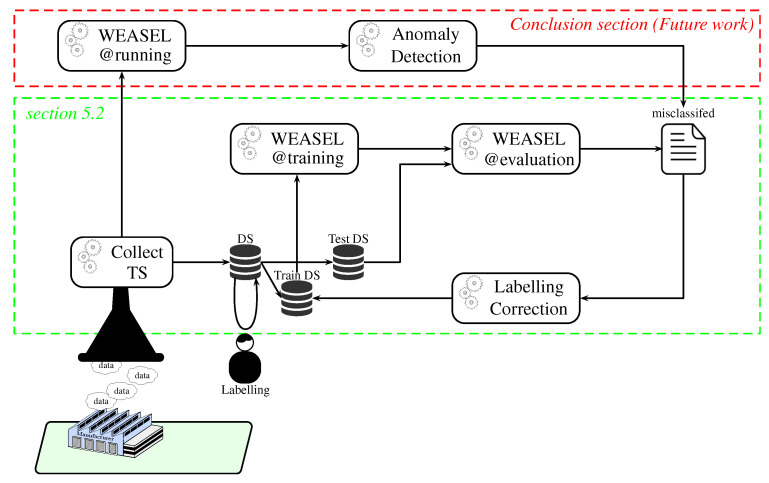
semi-automated (with Machine Learning) integration process.

Before being able to use WEASEL@running for classifying new parts (and, therefore, sorting them out), the use of ML requires collecting time series as datasets for training and evaluating our models (cf. Figure 4). Although the data collection, the training, and the evaluation processes can be automated, a human intervention is still needed to label our data (i.e., assigning the right class/colour at each time series). In the same way, and according to the accuracy of our model, misclassifications of the test TS can be re-labelled for re-training the model and improving it.

This pipeline has been evaluated on our testbed. The next section provides the results and discussions.

## 5. Models Evaluation

**Figure 5:**
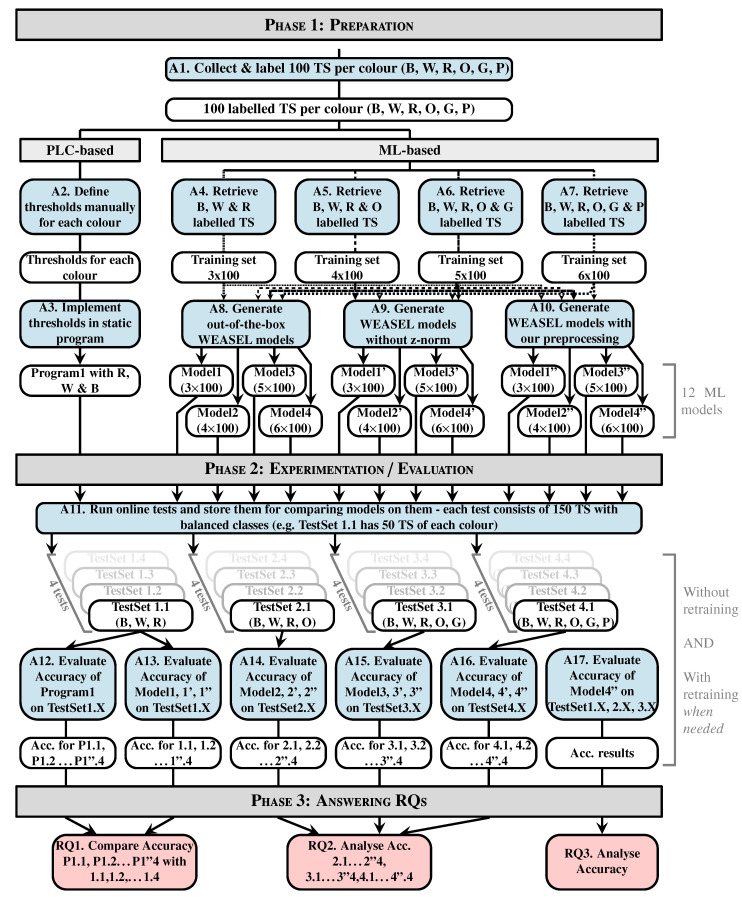
Methodology for models preparation, creation, and evaluation: The blue boxes correspond to the activities, the white ones to the input/output, the grey ones to the different phases, and the pink ones to the analyses for answering the research questions.

Figure 5 presents the methodology defined for conducting our experiments and evaluations. Additionally, all of our experiments are conducted by logging each TS and the corresponding class that is predicted by WEASEL on the controller, in order to save the testing data sets for offline replays, for example. In addition, parts have always been following a predetermined sequence, so as to know the ground truth and to be able to evaluate the results.

We evaluate the results while using the classification accuracy indicator, which is computed using Equation (1).

(1) $$Accuracy=\frac{number\;of\;correctly\;classified\;instances}{total\;number\;of\;instances}$$

Note that we do not use the other metrics, such as precision, recall, and F1-measure, since we have balanced datasets, which means that the classes inside of the datasets contain the same proportion of samples (e.g., in the three-colour test datasets, 33.3% of the parts are blue, another 33.3% of the parts are red and the last 33.3% are white, and the costs of misclassifications are the same whether they are false positives (i.e., for the blue class, an actual white or red part classified as a blue part) or false negatives (i.e., an actual blue part classified as a red or white part). In this specific case, the classification accuracy metric itself is meaningful and sufficient, while it may not be the case when the datasets are imbalanced and, when, for example, false negatives are more important than false positives, such as in the study of the propagation of a virus.

The study has been driven following three research questions (RQ), namely, RQ1: can WEASEL (off-the-shelf) be as accurate as manually set thresholds on verifying and sorting the three original colours? RQ2: Is WEASEL able to handle evolutions? RQ3: Is one single generic model accurate enough or is a model for each set of colours mandatory?

### 5.1. Preparation

Before evaluating both our static program and our models, the first imperative is to collect data. For each six colours we had (three originals—*blue, white, red*—and the three others we 3D printed—*orange, green, purple*—), 100 time series (TS) have been collected; this makes a total of 600 TS where the sizes range from 350 to 500 samples each. The labelling is done manually with classes between one and six. The first 100 TS are blue; therefore, the class assigned is one, the second 100 TS are white, so the class assigned is two, so on and so forth. This step corresponds to the first activity, denoted A1 in Figure 5.

Following the different steps of the program logic integration (Fetch TS, Analyse them, Build program, & Integrate it), thresholds have been defined. The definition of the threshold has been done based on the minimal value range, as depicted in Figure 2, except the 100 TS for each colour were used to determine the range. With these defined thresholds, the program logic that is shown in Algorithm 1 has been built. This type of logic is what is implemented in old static controllers. These steps correspond to activities 2 and 3, denoted A2 and A3 in Figure 5.

**Figure 6:**
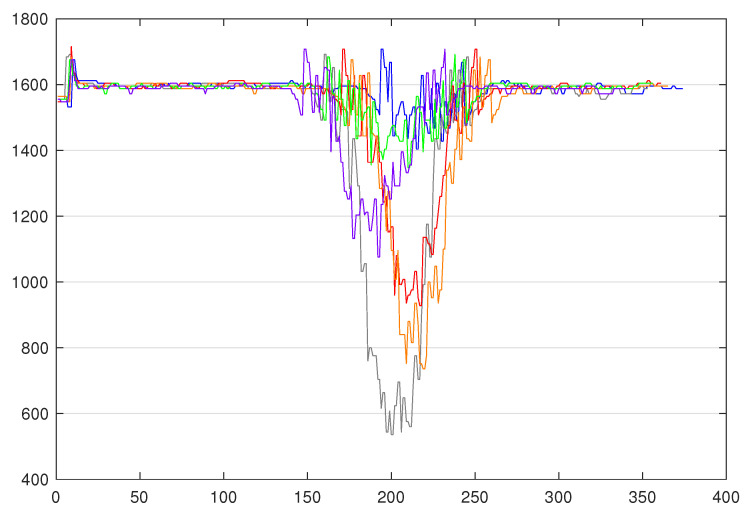
6 colours TS with no preprocessing.

**Figure 7:**
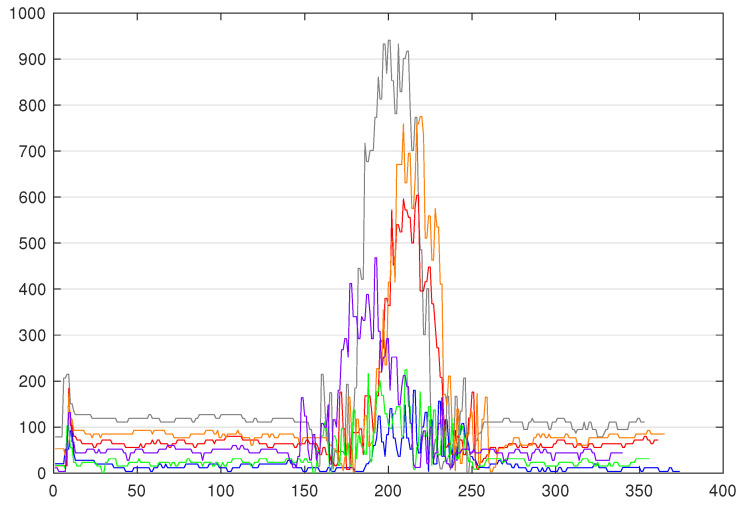
6 colours TS after preprocessing.

The next important step is to create the ML models. In order to represent the evolution of the production in terms of variety of product, we chose to create models trained on three colours, four colours, five colours, and six colours. In order to train such models, training datasets are created by fetching the labelled TS (A4 to A7 in Figure 5). Therefore, a total of four training datasets was obtained. The first one contains 100 TS per colour and three colours, so it is basically composed of 300 TS. The second one is the same as the 300 TS training set, in which we added the 100 TS of the fourthxs colour, so it is composed of 400 TS. The third one is the same as the 400 TS training set, in which we added the 100 TS of the fifth colour. Finally, the fourth one is the same as the 500 TS training set, in which we added the 100 TS of the sixth colour. For each set of colours, three types of models are generated, namely out-of-the box models (with default parameters) denoted *ModelX*, models without preprocessing denoted *ModelX’* and models with our preprocessing method denoted *ModelX”*. Assuming that WEASEL (out of the box) could be not effective enough in our case where the major differences between the TS reside in their amplitude (as shown in Figure 6) due to its intrinsic z-normalisation method, we investigate it with this normalisation disabled. As the TS amplitudes are relatively close between classes, we propose a preprocessing method, so as to separate them. This makes a total of 12 models that will be evaluated and compared:

- **Four out-of-the-box models** (cf. activity A8 in Figure 5): in order to observe how effective the out-of-the-box configuration is, four models were trained. As mentioned before, WEASEL natively uses the z-normalisation as a preprocessing method. This method ensures that the output vectors (TS) have a mean around 0 and standard deviation close to 1. That means that the output TS on which the models are trained will have the same range of amplitudes. *Model1* is a model that is trained with default parameters on the three-colour 300 TS data set. *Model1* took 17.0 s to train. *Model2* is trained on the four-colour 400 TS data set and took 64.9 s to train. Following this pattern, *Model3* is trained on the five-colour 500 TS data set and took 92.2 s to train, and *Model4* is trained on the six-colour 600 TS data set and took 112.6 s to train, still with default parameters.
- **Four no-preprocessing models** (cf. A9 in Figure 5): In order to verify whether the z-normalisation method is effective enough in a case where the major differences between the TS reside in their amplitude, four models were trained the same way, except the default z-normalisation applied in WEASEL has been disabled. This means that the models are trained on raw data. By doing so, the amplitude of the TS will become an important feature. Accordingly, *Model1’* is trained (without z-normalisation) on the same dataset as model *Model1* (3 × 100 TS, three colours), *Model2’* on the same dataset as *Model2* (4 × 100 TS, four colours), *Model3’* on the same as *Model3* (5 × 100 TS, five colours) and *Model4’* on the same as *Model4* (6 × 100 TS, 6 colours). The elapsed times in the training process for *Model1’*, *Model2’*, *Model3’*, and *Model4’* are, respectively, 15.7 s, 24.5 s, 71.7 s and 96.0 s.
- **Four models with our preprocessing** (cf. activity A10 in Figure 5): For the purpose of concluding on the importance of a suitable preprocessing method, four models were trained with our preprocessing method. Let us first present the preprocessing method that we propose. This method is for ensuring the same reference for the TS inside of the same class. Because the means of the TS inside of the same class are the same, but they are different between each class, we chose the mean as the reference for the TS. Our preprocessing is as follows: (i) for each TS, we compute the mean value of the whole series, and subtract this mean value from each original element of the time series; (ii) for each modified element, we compute its absolute value, and replace the element by this absolute value (this reduces the brutal changes in the TS caused e.g., by the inaccuracy of the sensor measurement or any environmental change). Using this method, we not only ensure that TS from the same class have the same reference (their means), but we preserve the amplitude difference in the pattern and we also deliberately shift TS from different classes (as depicted in Figure 7 between y = 0 and y = 200, as opposed to y = 1600 in Figure 6), and the reference is also becoming a characteristic. We implemented this method in WEASEL’s code and trained the following models. *Model1”* is trained on the same training set as *Model1* and *Model1’* (3 × 100 TS, three colours), *Model2”* on the same training set as *Model2* and *Model2’* (4 × 100 TS, four colours), *Model3”* on the same training set as *Model3’* and *Model3* (5 × 100 TS, five colours), and *Model4”* on the same training set as *Model4’* and *Model4* (6 × 100 TS, six colours). The elapsed times in the training process for *Model1”*, *Model2”*, *Model3”*, and *Model4”* are, respectively, 17.6 s, 31.0 s, 157.6 s, and 243.4 s.

### 5.2. Experimentation-Evaluation

For evaluating the models and static program, testing data sets are needed. However, as already stated, the classification has to be done online, so we conducted an online evaluation of some of the models, and stored the testing data sets in order to compare the accuracies of different models on the same test sets. Overall, we gathered four series of four test sets, such as that depicted in Figure 5 (cf. A11). Each series of four test sets is composed by TS of different colours, i.e., the first series (*TestSet1.1* to *TestSet1.4*) is composed of TS of three colours, the second series (*TestSet2.1* to *TestSet2.4*) contains TS of four colours, the third series (*TestSet3.1* to *TestSet3.4*) contains five colours, and the fourth series (*TestSet4.1* to *TestSet4.4*) contains six colours. Each test set, regardless of the number of colours, is made of 150 TS, with balanced classes.

The first actual experiments (to answer RQ1) are the evaluation of the static program, *Model1*, *Model1’*, and *Model1”* (cf. A12 and A13 in Figure 5) on *TestSet1.1* to *TestSet1.4*. The accuracies are detailed in Table 2. By looking at those results, we can see that the static program is efficient and the native z-normalisation is not the best normalisation for a case where TS from different classes have the same patterns, but different amplitudes, since the z-normalisation normalises the TS, such that they have the same amplitude. *Model1* still gives satisfying results in this case.

The next experiment is the evaluation of *Model2*, *Model2’*, and *Model2”* (cf. A14 in Figure 5) on *TestSet2.1* to *TestSet2.4* (4 colours). The accuracies are presented in Table 3. Here, we can clearly see that the z-normalisation (*Model2*) is not optimal and our preprocessing (*Model2”*) gives satisfying results.

**Figure 8:**
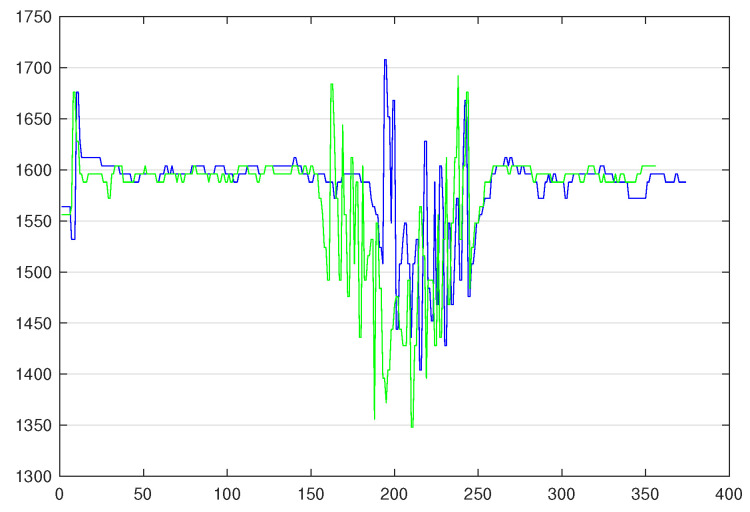
Blue and green time series.

The next experiment is the evaluation of *Model3*, *Model3’*, and *Model3”* (cf. A15 in Figure 5) on *TestSet3.1* to *TestSet3.4* (5 colours). The accuracies are presented in Table 4. With these results, we can see that neither the z-normalisation (*Model3*) nor no normalisation (*Model3’*) are optimal. This is due to the fact that some TS of different classes have the same amplitude, e.g., blue and green TS, as shown in Figure 8. However, our preprocessing method, since it adds an offset between TS of different classes, allows for WEASEL to classify TS with a better accuracy.

However, updating the training set with misclassified TS from previous tests and retraining the model allows to increase the accuracy. With *Model3’*, we took the misclassified TS from *TestSet3.1*, we corrected the label, and then added them to the training set, so the set contains 581 TS. Subsequently, we replayed the test on *TestSet3.2* with the updated *Model3’*, and we reiterated that the retraining process (the full process is shown in Figure 4—green box). *Model 3’* is now trained on 587 TS, and the accuracies are shown in Table 5.

The next activity (A16 in Figure 5) is the evaluation *Model4*, *Model4’*, and *Model4”* on *TestSet4.1* to *TestSet4.4* (6 colours). The accuracies are presented in Table 6. In this scenario, we can see that the models with or without z-normalisation (i.e., *Model 4 and 4’)* give very low accuracies and a large deviation between the test sets can be observed. By contrast, *Model4”* accuracies are close to satisfactory results, and there is room for improvement with retraining.

*Model4”* has therefore been retrained using exactly the same process as previously. We relabelled the misclassified TS from the test on *TestSet4.1*, added them to the training set which now contains 608 TS and retrained *Model4”*. Then we ran a test on *TestSet4.2* with the updated *Model4”*, and took the misclassified TS from this test, relabelled them and updated the training set a second time. *Model4”* has been retrained on the updated training set containing 621 TS, and we conducted tests on *TestSet4.3* and *TestSet4.4*. The accuracies are shown in Table 7. We can see that retraining the model increases the accuracy on the test with *TestSet4.3*, but slightly decreases it on the test with *TestSet4.4*.

The final experiment (A17 in Figure 5) consists of the evaluation of *Model4”* (since it is the one with the best results) on all of the test sets, except the ones with six colours, since it has already been done. This experiment aims at verifying whether it is possible to train only one “complete” model accurate enough to correctly classify parts, even if some sort of parts are missing, e.g., the model is trained to classify products x, y, and z, but z is out of stock for some time, is it possible to keep the xyz model or is a xy model needed? Detailed accuracies are given in Table 8. All of the results are satisfactory.

### 5.3. Answering RQs

Based on the results that are presented in the previous subsection, RQs can now be answered.

First, **RQ1** is the following: can WEASEL (off-the-shelf) be as accurate as manually set thresholds on verifying and sorting the three original colours? The off-the-shelf WEASEL models we tried are not as accurate as the static program on classifying the original three colours; however, they still give satisfying results. On the other hand, WEASEL models without z-normalisation or with our preprocessing method are as accurate as the static program.

Second, **RQ2** is the following: is WEASEL able to handle evolutions? WEASEL is able to handle evolutions in terms of number of different colours/parts. However, out-of-the-box models may not be the most accurate when adding more parts, and the preprocessing method used has a strong impact on the results. Finding the most suitable preprocessing method (as we did) is needed in order to obtain the best accuracy possible. The results can still be improved by updating the training set and retraining the model.

Finally, **RQ3** is the following: is one single generic model accurate enough or is a model for each set of colours mandatory? According to these results, it is possible to use a single model that can accurately classify parts, even if some of the colours are included in the training set, but not in the testing set. In this case, that means that we do not need to generate a model per range of parts, which means that there is no reconfiguration to be done if the line needs to produce/sort/verify a sub part of the entire range that it can produce/sort/verify. Note that this may only be true for unidimensional feature spaces, high dimensional feature spaces cases may behave differently.

## 6. Discussion

In the previous sections, we demonstrated that a specific TSC method, namely WEASEL, which is integrated into an automation architecture, is able to enhance the flexibility of the production lines. Indeed, we showed that: (i) the TSC method can be as accurate as a static program, so the effectiveness of the classification is not decreased; (ii) the TSC method is able to handle changes in terms of number of different colours/parts with some adaptations to the preprocessing method used before training the model; and, (iii) a single model trained on all of the parts can only classify a sub-part of the total variety of products. Of course, some kind of expertise is required in order to successfully apply such a method, especially with the preprocessing method, and there are limits and threats to validity, which are discussed in this section.

First, we chose to train the models with the maximum number of TS that we had for each colour. It might not be the most efficient method, since, for example, for the three colours model (blue, white, red), WEASEL without normalisation only needs 10 TS of each colour to reach 100% average accuracy on the four experiments. However, for the four and five colours experiments, the model trained on 100 TS of each colour is the most accurate.

Additionally, we could observe the colour sensor limitations, when considering that the range of values that it can return is quite narrow (0–2000 mV), it may not be possible to add more colours, because the TS corresponding to these colours will overlap (see when there is only one TS of each colour for the six colours in Figure 6). Note that, nonetheless, it also occurs in our experiments, in particular with blue and green colours. That is the reason why (i) it is really difficult to manage in a static program (where thresholds need to be defined) and (ii) the ML-based program was experimented.

Furthermore, we observed that a single model generated with all the data (six colours) is accurate, even when classifying only three, four, or five colours. This allows efficiency gains, since the same model can be used at all time, there is no need to change the model if some parts are not produced/sorted/verified for some time. However, as the classification is done on-the-fly, it may be important to look at the impact of the model size on the prediction time. If the model size increases, the prediction time to the point where the classification result is given so late that the controller cannot send the instruction to the actuator to push the part before the part is in the front of the right stock, then it is better to use a smaller model if the variety of parts is reduced (even temporarily).

Note that, even though an experimental evaluation of the prediction time has not been detailed in this paper, online tests allowed for us to witness that all of the parts have been sorted on time, which means that the prediction time is suitable.

Finally, regarding the preprocessing methods we investigated, we could observe that the z-normalisation is not suitable in our case, since the amplitudes of the TS need to be retained for the TSC method to correctly classify them. We also observed that training the models on raw data is not suitable either. When applying our proposed preprocessing method, we witness that the models’ training time increases exponentially with the number of TS in the training set, while it increases linearly with the other two methods. However, our preprocessing method allows for accurately classifying the parts. Note that the training time is not as important as the prediction time in this case study, which was not impacted by the preprocessing method.

Additionally, we are aware that the degradations we could observe under these conditions while using the WEASEL method could have been different with another TSC method. However, the adaptations that we propose in terms of data preprocessing before training models could be suitable for other methods.

Overall, using ML-based methods instead of static programs presents numerous advantages and plays an important role in the improvement of the flexibility of production lines. Some of these advantages are the following:

- ML-based methods allow for distinguishing patterns in time series, even when a human and, thus, a static program cannot distinguish them
- They allow for changing the variety of parts to be produced without having to change the program and, consequently, without having to stop the production line (provided that the model has been trained on data for all parts, obviously)
- They allow for processing a large amount of data

However, we could notice that these methods are not flawless. Indeed, they also have some drawbacks:

- The methods require expertise to tune them; they are not accurate enough out of the box
- Decisions can be made too late if the method is not fast enough, since they are made on a remote agent (request and response transmission time, and remote code execution time have to be considered)
- Some of the methods require several days to build a model (e.g., DTW-based methods on a lot of data)

## 7. Conclusions

In order to improve *flexibility* in today’s static industries and help the manufacturers to optimise the Overall Equipment Effectiveness, we proposed using a ML-based method instead of static programs and demonstrated the feasibility of applying such a method in the industrial context based on a scale model of a factory. This method gives promising results and allows for increasing the range of coloured parts to be sorted on the same sorting line when it would be impossible to manually set thresholds to distinguish two colours. Indeed, we extended the use of WEASEL in order to apply it on time series with almost the same patterns and only the amplitude differing, but this requires some expertise and comes at the cost of a reduced robustness against environmental changes. In order to tackle this robustness reduction problem, we proposed modifying the preprocessing method used in WEASEL. Finally, we showed that, in our case, the production line could be even more flexible and uses only one model whether it is to produce/verify/sort the complete variety of parts or only a sub part.

The way that we implemented the WEASEL method might not be the best, even when disabling the z-normalisation method, because it has a negative impact on the robustness, and we had to propose another preprocessing method in order to mitigate the impact. Additionally, we faced a few issues on such a simple canonical case; issues might be larger and more complex when applying such a method on real manufacturing processes.

For future research, we will consider the automation of the retraining process, as our use case is based on the colour verification and sorting of coloured parts, we know, *a priori*, the sequence of parts that have to be sorted. We will also take a closer look at prediction times, especially when we use a complete model for classifying a sub part of the whole variety of parts. Moreover, we will explore whether the ML-based method is also able to handle changes in the production speed. Finally, we will add some security mechanisms, so as to build a trusted complete framework, as described in Figure 4.

## Figures and Tables

**Table 1:** Implementation of time series classification (TSC) on industrial cases.

Paper	Approach		Method	TS Type		Domain	Application
	Trad.	DL		Uni.	Multi.		
[40]		X	LSTM autoencoder + Deep FeedForward Neural Net.		X	Process planning	Quality defects detection
[41]		X	Multi-Layer Perceptron		X	Transportation	Driver identification
[42]	X		Decision trees, SVM, kNN, Random Forests		X	Smart buildings	Energy quality classification
[43]	X		ED, FR, DTW, MDTW + similarity forests	X		Telecom.	Churn detection
[44]		X	Multi-Layer Perceptron		X	Gaming industry	Bot detection
[45]	X		wDTW + 1-NN	X		Sport	Pitcher’s performance analysis
[46]		X	Deep Neural Net.		X	Manufacturing	Lithium-ion cell selection
[47]	X		MASS + kNN		X	Agriculture	Chicken welfare assessment
[48]	X		Their method (Clustering + similarity tree)	X		Manufacturing	Self-learning + classification
[49]		X	Deep Belief Net. for feature leaning + LSTM		X	Health	Sleep patterns classification
[50]	X		Random Forests		X	Electrical industry	Short-term voltage stability assessment
[51]	X		Principal Component Analysis + Bayesian Neural Net.		X	Climatology	Atmospheric new particle formation prediction
[52]	X		Statistical feature extraction + Bayesian classifiers		X	Manufacturing	Robot execution failures classification
[53]		X	Convolutional Neural Net.		X	Manufacturing	Tool wear prediction
[54]		X	Convolutional Neural Net.		X	Transportation/safety	Drivers’ emotions/behaviour classification
[55]		X	Long-term recurrent convolutional LSTM		X		Pattern recognition and forecasting
[56]		X	Convolutional Neural Net.		X	Cosmology	Supernovae classification
[57]	X	X	Random Forests (baseline), LSTM-RNN, MS ResNet, TempCNN		X	Satellite imagery	Vegetation modeling and crop type identification
[37]	X		Their method (clustering)	X	X	Manufacturing/Power grid	Anomaly detection
[58]	X		Wavelet decomposition + SVM		X	Manufacturing	Machining condition monitoring
[59]		X	LSTM		X	Manufacturing/Prognostics	Remaining useful life prediction
[60]		X	Convolution recurrent Neural Net. (CNN + LSTM)		X	Manufacturing/Prognostics	Bearings health indicator construction
[61]	X		kNN		X	Manufacturing/Prognostics	Remaining useful life prediction
[62]	X		Random Forests, AdaBoost, CART, RR, SVR, RVFL		X	Additive manufacturing	Surface roughness prediction

**Table 2:** Classification accuracies of the static program and the models on three colours.

Test Set	1.1	1.2	1.3	1.4
Static Program	100%	100%	100%	100%
*Model1*	99.3%	100%	99.3%	96.7%
*Model1’*	100%	100%	100%	100%
*Model1”*	100%	100%	100%	100%

**Table 3:** Classification accuracies of the models on four colours.

Test Set	2.1	2.2	2.3	2.4
*Model2*	93.3%	86.7%	86.7%	81.3%
*Model2’*	100%	100%	100%	100%
*Model2”*	98.7%	96.7%	97.3%	99.3%

**Table 4:** Classification accuracies of the models on five colours.

Test Set	3.1	3.2	3.3	3.4
*Model3*	64.7%	60.7%	66.7%	80.7%
*Model3’*	46%	40%	40%	44%
*Model3”*	98%	98.7%	98%	99.3%

**Table 5:** Classification accuracies of Model3’ after the retraining process.

Test Set	3.1	3.2	3.3	3.4
*Model3’*	46%	40%	40%	44%
*Model3’* updated once (581 TS)	/	96%	*nn*	*nn*
*Model3’* updated twice (587 TS)	/	/	97.3%	100%

/ means test not done since TS used to retrain the model. *nn* means not needed.

**Table 6:** Classification accuracies of the models on six colours.

Test Set	4.1	4.2	4.3	4.4
*Model4*	52.7%	22.6%	35.3%	74%
*Model4’*	24.7%	31.3%	20%	33.3%
*Model4”*	94.7%	79.3%	79.3%	100%

**Table 7:** The classification accuracies of Model4” after the retraining process.

Test Set	4.1	4.2	4.3	4.4
*Model4’*	94.7%	79.3%	79.3%	100%
*Model4”* updated once (608 TS)	/	91.3%	*nn*	*nn*
*Model4”* updated twice (621 TS)	/	/	95.3%	98.7%

/ means test not done since TS used to retrain the model. *nn* means not needed.

**Table 8:** Classification accuracy of Model4” on three, four, and five colours.

Test Set	1.1	1.2	1.3	1.4	2.1	2.2	2.3	2.4	3.1	3.2	3.3	3.4
Model4”	100%	100%	100%	100%	98.7%	96.7%	98%	99.3%	97.3%	98%	97.3%	100%
